# A randomized controlled trial for comparing efficacy and safety between intraarticular polynucleotide and hyaluronic acid for knee osteoarthritis treatment

**DOI:** 10.1038/s41598-023-35982-z

**Published:** 2023-06-09

**Authors:** Tae Woo Kim, Moon Jong Chang, Chung Yeop Shin, Chong Bum Chang, Seung-Baik Kang

**Affiliations:** 1 Department of Orthopedic Surgery, Seoul Metropolitan Government-Seoul National University Boramae Medical Center, 20, Boramae-ro 5-gil, Dongjak-gu, Seoul, 07061 South Korea; 2 Department of Orthopaedic Surgery, Seoul National University Bundang Hospital, Seongnam, South Korea; 3 Department of Orthopedic Surgery, Seoul National University College of Medicine, Seoul, South Korea

**Keywords:** Medical research, Rheumatology

## Abstract

Although the use of intra-articular polynucleotide (IA PN) injection as a viscosupplement for knee osteoarthritis (OA) treatment has been proposed, its efficacy and safety compared to high molecular weight hyaluronic acid (HMWHA) injection has not yet been established. The present double-blind, multicenter, randomized controlled trial aimed to investigate the efficacy and safety of IA PN injection compared to IA HMWHA injection. A total of 60 patients (15 men, 45 women, 64.5 ± 7.5 years) with knee OA (Kellgren–Lawrence grade 1–4) were randomly allocated to each group. All patients were given three IA injections of PN (n = 30) or HMWHA (n = 30) at intervals of 1 week. The primary endpoint was the change rate in weight-bearing pain (WBP) 16 weeks from the baseline. The secondary endpoint included multiple measurements: the change rate in WBP rate at 8 weeks; the change rate in pain level at rest and during walking at 8 and 16 weeks; the Korean-Western Ontario and McMaster University Osteoarthritis index; the Euro-Quality of Life-5 Dimension; Clinical Global Impression, Patient Global Impression at 8 and16 weeks, and total consumption of rescue medicine. The mean change rate in the WBP at 16 weeks from the baseline was − 54.0 ± 38.1% in the IA PN group and − 42.8 (± 35.8%) in the IA HMWHA group, and there was no significant difference between the two groups (*p* = 0.296). All secondary endpoints related with pain and functional outcome also showed no significant difference between the two groups. Pain at the injection site and swelling were reported as adverse events, and the incidence was similar between the two groups. IA PN showed comparable efficacy and safety to IA HMWHA at 3 times injection with an interval of 1 week. IA PN can be useful alternative to IA HMWHA for the treatment of knee OA.

## Introduction

Intra-articular (IA) injection therapy has an important role in the treatment of osteoarthritis, especially in patients with insufficient response to medication or comorbidities that restrict medical treatment^1–4^. Intra-articular hyaluronic acid (IA HA), which is used for viscosupplementation in the synovial joint, has been widely used for the treatment of osteoarthritis (OA), and numerous studies support its clinical efficacy and safety^5–8^. However, recent OA treatment guidelines based on the results of high-quality, unbiased studies, report a lack of generalized effect as a limitation of IA HA treatment^1,3,9–14^. In some patients, pseudoseptic arthritis symptoms such as painful swelling and redness have been reported after IA HA use^14,15^.

Intra-articular polynucleotide (IA PN) has been proposed as an alternative of IA HA for viscosupplementation over the past decade^16–20^. PN is a polymeric molecule of long-chain DNA fraction with a high molecular weight (MW) extracted from the testes and sperms of salmons. Its ability to bind to a large amount of water provides viscoelasticity in the joint space and can be used as a supplement for OA treatment^21^.

Several studies have compared the efficacy and safety of IA PN and HA, and IA PN has shown comparable or superior clinical outcomes than those of IA HA. Vanelli et al^16^. and LS. Giarratana et al^21^. reported that pain reduction and clinical score improvement was comparable between the IA PN, and IA HA groups. In Zazgyva et al. study^22^, significant pain reduction was observed in both the IA PN and IA HA groups; however, a significant improvement in the Knee Osteoarthritis Score (KSS) was observed only in the IA PN group.

However, previous comparative studies have mainly used only low or medium MW HAs, and studies that have used IA HMWHA or cross-linked HA as a control for IA PN are very limited. Considering that many studies have reported the superior efficacy of IA HMWHA compared to low-or medium MWHA^23,24^, existing data associated with IA PN and IA HA use are not sufficient to support the clinical use of IA PN as an alternative of IA HMWHA, which is widely used currently.

Therefore, the present randomized controlled study aimed to investigate the efficacy and safety of IA PN compared with IA HMWHA in the treatment of knee OA. We hypothesized that IA PN could be a useful alternative of HMWHA in the treatment of knee OA.

## Material and methods

### Products

The PN used in this clinical study was Conjuran® (PharmaResearch, Gangneung-si Kangwon-do, Republic of Korea). A prefilled syringe containing 2 ml of viscoelastic PN solution 20 mg/ml that was extracted from salmon. It was approved as a medical device in Korea for physical viscosupplementation in patients with knee OA. The HA used in this study was Hyruan Plus^®^ (LG Life Science, Iksan, South Korea), a linear HMWHA with a mean MW of 3000 kDa, and its clinical efficacy and safety have been well established in previous studies^6^.

### Study design

The present randomized, double-blind, multicenter (two investigational sites) study was conducted from January 2020 to March 2021. After patient screening and a wash-out period of two weeks, 60 patients with symptomatic knee OA were randomly allocated to each group (PN or HMWHA) using the block randomization method (Microsoft Excel^®^) in a 1:1 ratio. To ensure a double-blind condition, the patients and investigators were concealed from the group assignment. The injection was administered by an independent physician who was not blinded to the injection product, and a blinded investigator performed the clinical assessment. Informed consent was obtained from all the patients enrolled in this study. This clinical trial was conducted in accordance with the principles of the Declaration of Helsinki and in good clinical practice. Institutional review board of each institute (Seoul National University Boramae Medical Center Institutional Review Board, and Seoul National University Bundang Hospital Institutional Review Board) approved the study and it was registered in the Clinical Research Information Service Protocol Registration System (Trial number: KCT0008003, 13/12/2022). All methods were performed in accordance with the relevant guidelines and regulations.

### Study subjects

Sixty patients with symptomatic knee OA diagnosed based on the American College of Rheumatology Classification^25^ were enrolled in the present clinical trial. Additional inclusion criteria were as follows: (1) insufficient response to pharmacological treatment or physical treatment more than 3 months, (2) Kellegren–Lawrence (K–L) grade^26^ I–IV, (3) age 40 years or older, and (4) 40 mm or more weight-bearing pain (WBP) on a 100 mm visual analog scale (VAS) in at least one of the knee joints. Exclusion criteria were as follows: (1) history of trauma, (2) rheumatoid arthritis or metabolic arthritis, (3) infection of the affected joint, (4) previous surgery of the affected limb, (5) other conditions accompanying severe pain such as Paget’s disease, complex regional pain syndrome, and intervertebral disc herniation; (5) IA HA injection within 6 months or IA steroid injection within 3 months from the baseline; (6) use of anticoagulants or antiplatelet drugs except for low dose of aspirin (≤ 300 mg/day); (7) use of muscle relaxants and anti-inflammatory drugs within 2 weeks from the baseline; (8) physical therapy including herbal treatment, heat treatment, and acupuncture within 2 weeks from the baseline; (9) history of alcohol or drug abuse/dependence; (10) pregnant women or fertile women and men who have a pregnancy plan; (11) hypersensitivity to the components of medical devices used in the clinical research of this study.

### Interventions

**Figure 1:**
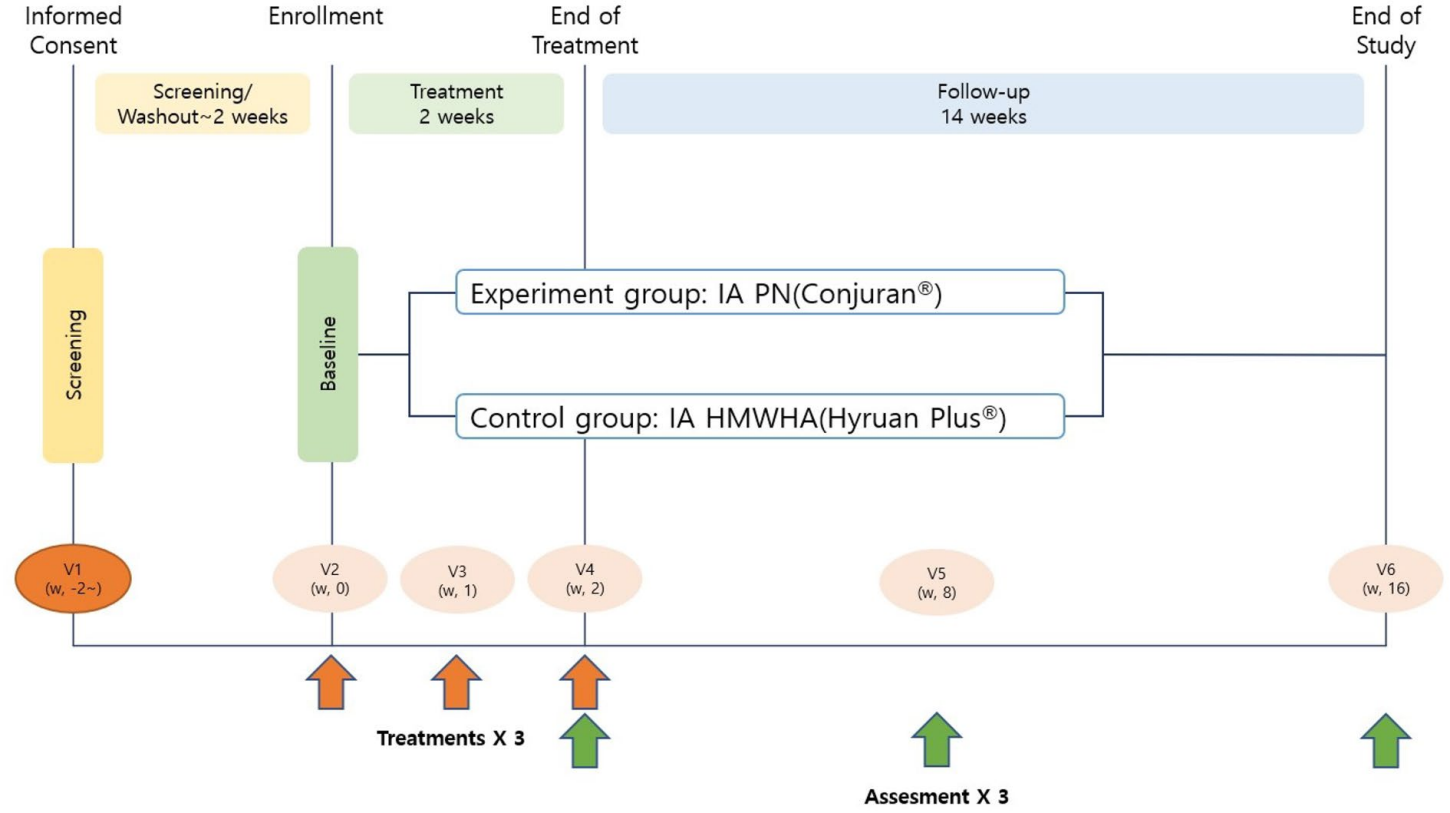
Study flow diagram.

All patients received three IA PN (20 mg/ml) injections or three IA HMWHAs (20 mg/2 ml) injections at intervals of 1 week. In each institute, the injection was aseptically administered by an independent, skillful orthopedic surgeon. The first injection was administered at the beginning of the treatment (baseline), and the second and third injections were administered at 1 week and 2 weeks from the baseline, respectively. After three IA injections, patients were followed up at 8 weeks and 16 weeks for clinical evaluation. During the study period, use of Acetaminophen (≤ 4 g/day) were allowed for the pain rescue drug (Fig. 1).

### Outcome measurements

The primary endpoint of this study was the VAS (100-mm) change rate for WBP at 16 weeks compared with baseline. The secondary endpoints were as follows: (1) VAS (100-mm) change rate for WBP at 8 weeks compared to baseline; (2) VAS (100-mm) changes in WBP at 8 and 16 weeks compared to baseline; (3) VAS (100-mm) change, and rate of change in resting and walking pain at 8 and 16 weeks compared to baseline; (4) the rate of change in Korean Western Ontario and McMaster Universities Osteoarthritis Index (K-WOMAC) scores at 8 and 16 weeks compared to baseline; (5) improvement of Clinical Global Impression (CGI), and Patients Global Impression (PGI) at 8 and 16 weeks compared to baseline; (6) evaluation of quality of life (EQ-5D) at 8 and 16 weeks compared to baseline, and (7) consumption of rescue medicine (acetaminophen) after visiting baseline. For safety analyses, all systemic and local adverse events (AEs) data were collected from the safety set population, and their severity and relationship with study intervention data were analyzed. Treatments for the management of AEs and AEs leading to the discontinuation of the study were also evaluated. The vital signs of patients were also evaluated at every visit.

### Statistical analysis

The sample size was calculated using an a priori power analysis. Based on previous literature that reported mean VAS difference of 1.7 mm with a standard deviation of 1.8 mm at 16 weeks after IA PN injection (α = 0.05, β = 0.8), it was expected that at least 19 cases were required for each group. Anticipating possible loss, 30 patients were enrolled in each group. Statistical analysis was conducted using SPSS version 25.0 (IBM, Armonk, NY, USA), and *p* < 0.05 was considered statistically significant. Depending on the data normality, Student’s t-test or Wilcoxon signed-rank and rank sum tests were used to evaluate intergroup differences in continuous variables. Categorical variables were analyzed using Pearson’s chi-squared or Fisher’s exact tests.

## Results

**Figure 2:**
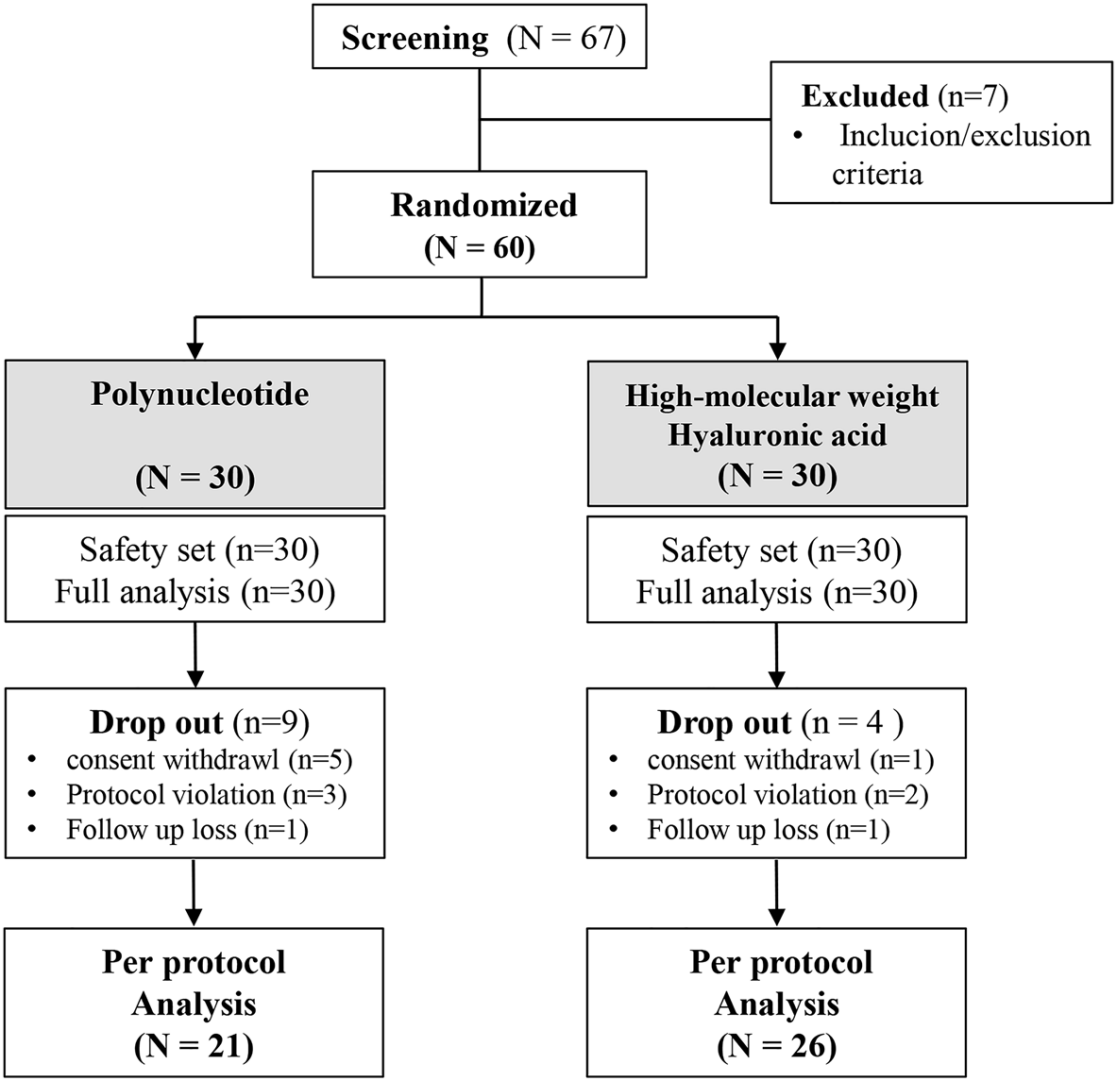
Flow chart of patient enrollment.

After screening 67 patients, 60 patients were randomly allocated into the study groups, and 47 patients completed the study (IA PN = 21, IA HMWHA = 26) (Fig. 2). Six patients were excluded due to lack of primary outcome assessment, five patients were excluded due to the use of contraindicated drugs, and two patients were excluded due to follow-up loss. Demographic variables including age, sex, smoking, drinking, K–L grade, and combined medical comorbidities showed no significant difference between the two groups (Table 1).

**Table 1:** Demographic and baseline characteristics.

	IA PN (n = 30)	IA HMWHA (n = 30)	*p*-value
Age	63.6 ± 6.7	65.4 ± 8.2	0.364 (t)
Sex			0.136 (c)
Male	5 (16.7)	10 (33.3)	
Female	25 (83.3)	45 (75.0)	
Kellgren–Lawrence grae, n (%)			0.614 (f)
Grade I	0 (0.0)	1 (3.3)	
Grade II	12 (40.0)	8 (26.7)	
Grade III	16 (53.3)	18 (60.0)	
Grade IV	2 (6.7)	3 (10.0)	
K-WOMAC (baseline)	38.5 ± 19.5	39.3 ± 16.3	0.889 (t)
EQ-5D	11.4 ± 3.2	10.7 ± 2.6	0.404 (t)
Smoker	1 (3.3)	1 (3.3)	1.000 (f)
Drinking	6 (20.0)	2 (6.7)	0.254

*IA PN* Intraarticular polynucleotide, *IA HMWHA* Intraarticular high molecular weight hyaluronic acid, *SD* standard deviation.

Testing for coutinuous variables between-treatment groups (two sample t-test ).

Testing for categorical variables between-treatment groups (Pearson’s chi-square test (c) or Fisher’s exact test (f)).

### Primary endpoint

**Figure 3:**
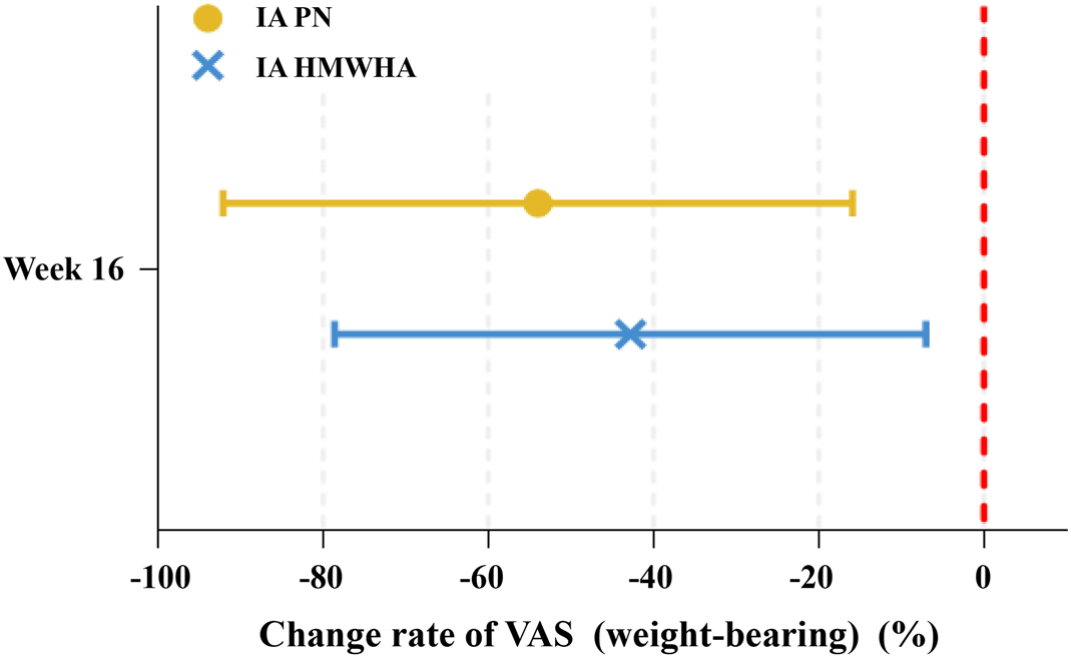
Comparison of change rate of weight-bearing VAS between IA PN and IA HMWHA at week 16. Significant reduction of weight-bearing VAS was observed from the baseline in both groups. However, there were no significant difference in change rate of weight-bearing VAS between two groups (IA PN = − 54.0 ± 38.1%, IA HMWHA = − 42.8 ± 35.8%, *p* = 0.296) *IA* Intraarticular, *PN* Polynucleotide, *HMWHA* High molecular-weight hyaluronic acid, *VAS* Visual analogue scale.

The VAS (100-mm) change rate for WBP from baseline to 16 weeks was − 54.0 ± 38.1% in the IA PN group, and − 42.8 ± 35.8% in the IA HMWHA group, and the IA PN group showed a higher VAS change rate than the IA HMWHA group. However, there was no statistically significant difference in the VAS change rate at 16 weeks between the two groups (*p* = 0.296) (Fig. 3). Both groups showed improvement in WBP at 16 weeks compared to baseline.

### Secondary endpoint

**Figure 4:**
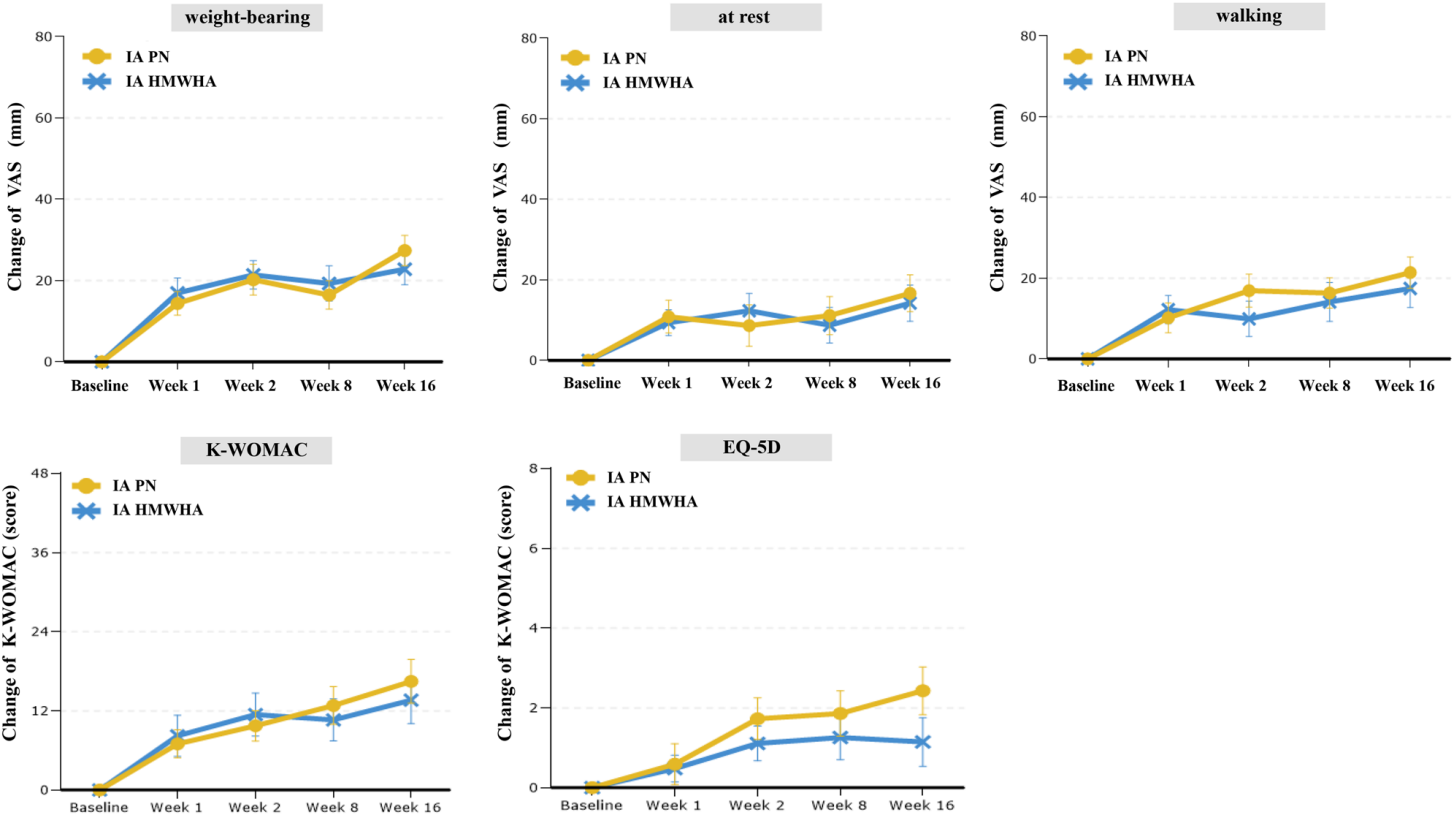
Comparative analysis of serial change of pain VAS, K-WOMAC, EQ-5D between IA PN and IA HMWHA during study period. Change of weight-bearing, at rest, and walking VAS, K-WOMAC, and EQ-5D scores were significantly increased from the baseline, and gradually increased until 16 weeks in both IA PN and HMWHA groups. However, there were no significant difference between two groups at all timepoints.

**Figure 5:**
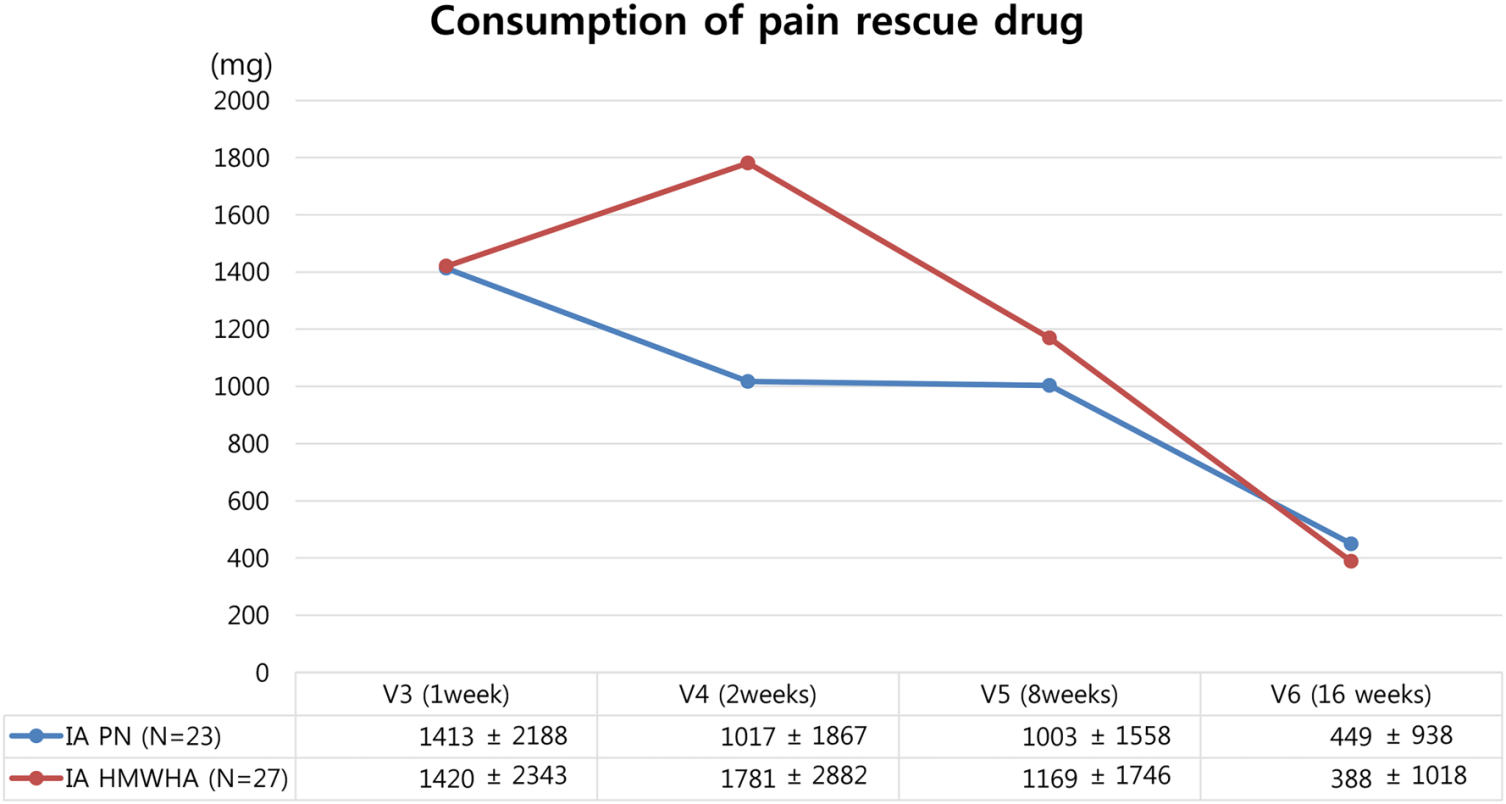
Consumption of pain rescue drug. Consumption of pain rescue drug in IA PN group decreased gradually until 16 weeks. There was no significant difference in consumption of pain rescue drug at every visits between IA PN and HMWHA groups.

All secondary endpoints related to pain VAS (100 mm) change and change rate for WBP, pain at rest, and walking pain at 8 and 16 weeks significantly improved from baseline, and VAS change gradually increased up to 16 weeks in both groups. However, there was no significant difference in any of the secondary endpoints related to pain VAS change between the IA PN and IA HMWHA groups (Table 2). The K-WOMAC change rate and EQ-5D, CGI, and PGI scores at 8 and 16 weeks also improved from baseline scores in both groups. However, there was no significant difference in any of the clinical scores between the IA PN and IA HMWHA groups (Table 3). Pain reduction and functional improvement were rapidly observed at two weeks from baseline, and clinical effects were sufficiently maintained until 16 weeks from baseline in both the IA PN and HMWHA groups (Fig. 4). The consumption of pain rescue drug (acetaminophen) at every visit was also similar between the two groups (Fig. 5).

**Table 2:** Comparison of Outcomes associated with pain VAS between IA PN and IA HMWHA groups.

	IA PN (n = 22)	IA HMWHA (n = 27)	*p*-value
Weight bearing VAS change rate			
Week 8—baseline (%)	− 33.9 ± 35.8	− 36.0 ± 40.2	0.847
Week 16—baseline (%)	− 54.0 ± 38.1	− 42.8 ± 35.8	0.296
At rest VAS change rate			
Week 8—baseline (%)	− 38.4 ± 73.7	− 26.6 ± 67.9	0.610
Week 16—baseline (%)	− 63.6 ± 76.3	− 60.5 ± 55.8	0.890
Walking VAS change rate			
Week 8—baseline (%)	− 28.6 ± 60.9	− 20.7 ± 55.0	0.639
Week 16—baseline (%)	− 41.2 ± 45.3	− 28.3 ± 51.2	0.374
Weight bearing VAS change (100 mm)			
Week 8—baseline (mm)	− 16.5 ± 16.5	− 19.3 ± 22.5	0.624
*p*-value (within)	0.000*	0.000*	
Week 16—baseline (mm)	− 27.4 ± 17.5	− 22.8 ± 19.8	
*p*-value (within)	0.000*	0.000*	
At rest VAS change rate (100 mm)			
Week 8—baseline (mm)	− 11.1 ± 22.1	− 8.7 ± 22.8	0.713
*p*-value (within)	0.028*	0.057	
Week 16—baseline (mm)	− 16.7 ± 21.1	− 14.2 ± 23.0	0.709
*p*-value (within)	0.002*	0.004*	
Walking VAS change rate (100 mm)			
Week 8—baseline (mm)	− 16.3 ± 17.6	− 14.1 ± 25.1	0.729
*p*-value (within)	0.000*	0.007*	
Week 16—baseline (mm)	− 21.4 ± 17.7	− 17.5 ± 24.2	0.538
*p*-value (within)	0.000*	0.001*	

*IA PN* Intraarticular polynucleotide, *IA HMWHA* Intraarticular high molecular weight hyaluronic acid.

*p*-value (within) : paired t-test, *p*-value : two sample t-test, *statistical significance (+).

**Table 3:** Comparison of clinical outcomes between IA PN and IA HMWHA groups.

	IA PN (n = 22)	IA HMWHA (n = 27)	*p*-value
K-WOMAC change			
Week 8—Baseline (score)	− 12.8 ± 13.4	− 10.6 ± 16.5	0.618
* p*-value (within)	0.000*	0.003*	
Week 16—Baseline (score)	− 16.5 ± 15.2	− 13.6 ± 18.0	0.566
* p*-value (within)	0.000*	0.001*	
EQ-5D change			
Week 8—Baseline (score)	− 1.9 ± 2.7	− 1.3 ± 2.9	0.454
* p*-value (within)	0.004*	0.032*	
Week 16—Baseline (score)	− 2.4 ± 2.7	− 1.2 ± 3.1	0.148
* p*-value (within)	0.001*	0.070	
CGI Score			
Week 8			
Very much improved	1 (4.5)	2 (7.4)	0.811
Much mproved	10 (45.5)	9 (33.3)	
Minimally improved	8 (36.4)	10 (37.0)	
No change	3 (13.6)	4 (14.8)	
Minimally worse	0 (0.0)	1 (3.7)	
Much worse	0 (0.0)	1 (3.7)	
Very much worse	0 (0.0)	0 (0.0)	
Week 16			
Very much improved	2 (9.5)	1 (3.8)	0.334
Much mproved	10 (47.6)	8 (30.8)	
Minimally improved	8 (38.1)	14 (53.8)	
No change	0 (0.0)	2 (7.7)	
Minimally worse	1 (4.8)	0 (0.0)	
Much worse	0 (0.0)	1 (3.8)	
Very much worse	0 (0.0)	0 (0.0)	
PGI Score			
Week 8			
Very much improved	1 (4.5)	0 (0.0)	0.433
Much mproved	6 (27.3)	10 (37.0)	
Minimally improved	12 (54.5)	10 (37.0)	
No change	3 (13.6)	4 (14.8)	
Minimally worse	0 (0.0)	2 (7.4)	
Much worse	0 (0.0)	1 (3.7)	
Very much worse	0 (0.0)	0 (0.0)	
Week 16			
Very much improved	2 (9.5)	1 (3.8)	0.434
Much mproved	10 (47.6)	8 (30.8)	
Minimally improved	6 (28.6)	13 (50.0)	
No change	2 (9.5)	3 (11.5)	
Minimally worse	1 (4.8)	0 (0.0)	
Much worse	0 (0.0)	1 (3.8)	
Very much worse	0 (0.0)	0 (0.0)	

*IA PN* Intraarticular polynucleotide, *IA HMWHA* Intraarticular high molecular weight hyaluronic acid, *K-WOMAC* Korean Western Ontario and McMaster Universities Osteoarthritis, *EQ-5D* Evaluation of quality of life, *CGI* Clinical Global Impression, *PGI* Patients global impression.

*Statistical significance (+).

### Adverse events

In local AEs, three patients showed knee pain in the IA PN group, and one patient showed knee swelling in the IA HMWHA group, and there was no significant difference between the two groups (*p* = 1.000) (Table 4). All four local AEs relieved without any treatment within a few days. Regarding systemic AEs, the IA PN group reported two serious AEs (one of diarrhea and one of hematochezia), and the IA HMWHA group reported one AE (hyperthyroidism). However, two serious AEs in the IA PN group had no causal relationship with the IA PN injection. None of the patients discontinued the study because of local or systemic AEs.

**Table 4:** Comparison of adverse events (AEs) between IA PN and IA HMWHA groups.

	IA PN (n = 30)	IA HMWHA (n = 30)	*p*-value
Local AEs			
Pain of knee	3	0	
Swelling	0	1	
Total	3	1	1.000 (f)
Systemic AEs			
Diarrhea serious AE(+) Relationship (none)	1	0	
Hematochezia serious AE (+) Relationship (none)	1	0	
Hyperthyroidism	0	1	
Total	2	1	1.000 (f)

*IA PN* Intraarticular polynucleotide, *IA HMWHA* Intraarticular high molecular weight hyaluronic acid.

Testing for categorical variables between-treatment groups (Fisher’s exact test (f)).

## Discussion

The principal finding of this study was that IA PN showed efficacy and safety comparable to IA HMWHA for the treatment of knee OA. Although statistical difference was not significant, the value of VAS (100-mm) change rate for WBP from baseline to 16 weeks was higher in IA PN group (− 54.0 ± 38.1%) than in IA HMWHA group (− 42.8 ± 35.8%). Clinical outcomes of the IA PN groups assessed by using K-WOMAC and EQ-5D were also comparable to that of the IA HMWHA group. All parameters associated with pain VAS and clinical outcomes significantly improved from baseline in both the IA PN and IA HMWHA groups.

Previous clinical trials that compared the analgesic efficacy between IA PN and IA HA have shown similar results. Vanelli et al^16^. and Zazgyva et al^22^. reported that the IA PN group showed similar pain VAS and knee injury osteoarthritis outcome score (KOOS) as the IA HA group at 16 weeks in randomized controlled trials. Meccariello et al^27^. showed that pain reduction and functional improvement were significantly higher in the IA PN group than in the IA HA group at 6-month follow-up in a retrospective study. However, previous studies have used only low- or medium-MW HAs ranging from 800 to 2000 kDa as controls for IA PN (Table 5). Several clinical trials have reported superior efficacy of HMWHA compared to low or medium MWHAs^23,24^; therefore, previous studies were not sufficient to support the alternative use of IA PN to IA HMWHA, which is recently widely used. These insufficient evidence can explain the reason why IA PN was not widely used for OA treatment for past decade.

**Table 5:** Clinical trials comparing clinical efficacy and safety between IA PN and HA in knee osteoarthritis.

Publication	Study Design	Treat	Control	Patients Number (Treat/Control)	Injection times	Results	
						Clinical efficacy	Safety
Current Study (Korea)	RCT	PN (Conjuran^®^)	HA (Hyruan Plus^®^) High MW (3000 kDa)	60 (30/30)	3	Reduced pain VAS/NSAID use, and improved K-WOMAC / EQ-5D in both group from the baseline No significant difference in all pain VAS, clinical scores, and rescue drug use between IA PN and IA HMWHA groups	PN: knee pain (n = 3) HA: knee swelling (n = 1) → natual relief Significant difference (−) No severe AEs
2014 (Italy)^21^	RCT	PN (Condrotide^®^)	HA (Hyalubrix^®^) Medium MW (1500–2000 kDa )	72 (36/36)	3	Reduced pain / NSAID consumption, and KOOS improvement in both group Significant KOOS “symptom” improve ment(PN : week 2 versus HA : week 18)	No significant AEs
2013 (Romania)^22^	RCT	PN (Condrotide^®^)	HA (Synocrom^®^) Medium MW (1600 kDa )	30 (15/15)	3	Reduced pain, and improved KOOS and KKS in both group from the baseline Significantly superior KKS improvement in PN group compare to HA group	Mild knee pain (PN: n = 2, HA : n = 1) → alleviated in a few hours No significant AEs
2013 (Italy)^27^	Retrospective	PN	HA Low MW	60 (30/30)	4		
2010 (Italy)^16^	RCT	PN (Condrotide^®^)	HA (Sinovial^®^) Low MW (800–1200 kDa )	60 (30/30)	5	Reduced pain VAS/NSAID use, and improved KOOS from the baseline in both group Similar trends in pain score between PN and HA groups	PN : mild knee pain (n = 1) → subsided within one hour

In the present study, IA HMWHA, with a mean MW of 3000 kDa, was used as a control for IA PN, and the results of this study support the use of IA PN as an alternative of IA HMWHA. Despite the well-established clinical efficacy of IA HA, recent OA treatment guidelines report a lack of generalized effects as a limitation of IA HA use^1,3^. When the clinical efficacy of IA HA is insufficient or IA HA shows an allergic reaction, IA PN can be a reliable alternative for viscosupplementation in the treatment of knee OA. In addition, recent studies reported that IA PN combined with IA HA can additionally improve pain VAS and clinical outcomes significantly compared to the single use of IA HA^28–30^. The combined use of IA HA and PN can be considered for synergistic clinical effects.

Another interesting finding of this study was that IA PN showed a fast onset of clinical efficacy and a sufficient duration of clinical effect. In this study, pain VAS during weight-bearing, at rest, and walking rapidly reduced within 2 weeks from the baseline and showed a similar pattern in the IA PN and IA HA groups. In Giarrantana et al. study^21^, IA PN showed significantly faster improvement only in KOOS (at 2 weeks) compared to IA HA (at 18 weeks). However, in our study, both the IA PN and HMWHA groups showed a rapid clinical improvement in all primary and secondary parameters. In addition, the clinical effect was maintained until 16 weeks from the baseline in all parameters associated with pain VAS and clinical scores including K-WOMAC and EQ-5D. Although IA corticosteroid injection for the treatment of knee OA has shown established clinical effects, a relatively short duration of action within 3 months has been suggested as an unresolved limitation^31,32^. In this clinical trial, IA PN showed the property of viscosupplementation that can compensate for the short-acting IA corticosteroid.

Regarding AEs related to treatment, minimal local AEs were reported (IA PN: three knee pain, IA HMWHA: one knee swelling) in this study, and local AEs spontaneously relieved without any treatment within a few days. Two systemic and serious AEs in IA PN were found to have no causal relationship with IA injections. The results of this study correspond well with those of previous studies that reported minimal AEs associated with IA PN administration, except for mild joint pain that resolved spontaneously within a few hours (Table 4).

Our study had several limitations. First, the number of enrolled centers, patients, and total sample size was relatively small, and therefore, a sufficient number of OA patients with diverse KL grades were not included, and the influence of OA K-L grade on the outcome of intra-articular injection was not evaluated. However, the number of patients in this study was calculated based on statistical power analysis, and there was no significant difference in KL grades between the two groups. Second, in this study, objective imaging assessment was not performed. However, numerous studies on clinical efficacy of intraarticular viscosupplementation such as hyaluronic acid, and polynucleotide mainly investigate pain, and clinical outcomes. It maybe because primary expectation for the use of IA viscosupplementation is pain relief and functional improvement rather structural improvement that can be evaluated with cartilage thickness on MRI or joint space narrowing on plain radiograph. Almost clinical decision to use or stop IAHA or IA PN in outpatient department also performed based on patient’s pain or functional improvement. Therefore, we believe that the results of this study can provide meaningful clinical information related with IA PN use. Third, the maximum follow-up period was 16 weeks from baseline, which was not sufficient for the evaluation of the long-term effects of each treatment. However, both IA PN and HMWHA groups showed a gradual increase in clinical effect until 16 weeks and these two IA viscosupplementations can compensate for the short-acting IA corticosteroids.

## Conclusion

IA PN showed comparable efficacy and safety to IA HMWHA at 3 times injection with an interval of 1 week. IA PN can be an useful alternative to IA HMWHA for the treatment of knee OA.

## Data Availability

Data described in this study will be made available upon request pending application and approval from the corresponding author.
